# Piloting an ICU follow-up clinic to improve health-related quality of life in ICU survivors after a prolonged intensive care stay (PINA): study protocol for a pilot randomised controlled trial

**DOI:** 10.1186/s40814-021-00796-1

**Published:** 2021-03-30

**Authors:** M. Rohr, S. Brandstetter, C. Bernardi, C. Fisser, K. P. Drewitz, V. Brunnthaler, K. Schmidt, M. V. Malfertheiner, C. J. Apfelbacher

**Affiliations:** 1 Medical Sociology, Institute of Epidemiology and Preventive Medicine, University of Regensburg, Dr.-Gessler-Str. 17, 93051 Regensburg, Germany; 2 University Children’s Hospital Regensburg, University of Regensburg, Klinik St. Hedwig, Steinmetzstr., 1-3, 93049 Regensburg, Germany; 3 Department of Internal Medicine II, University Hospital Regensburg, Franz-Josef-Strauss-Allee 11, 93053 Regensburg, Germany; 4 Institute of Social Medicine and Health Systems Research, Otto-von-Guericke University Magdeburg, Leipziger Str. 44, 39120 Magdeburg, Germany; 5 Institute of General Practice and Family Medicine, Charité University Medicine, Charitéplatz 1, 10117 Berlin, Germany; 6 Institute of General Practice and Family Medicine, Jena University Hospital, Bachstr. 18, 07743 Jena, Germany

## Abstract

**Background:**

Intensive care unit (ICU) survivors often suffer from cognitive, physical and mental impairments, known as post-intensive care syndrome (PICS). ICU follow-up clinics may improve aftercare of these patients. There is a lack of evidence whether or which concept of an ICU follow-up clinic is effective. Within the PINA study, a concept for an ICU follow-up clinic was developed and will be tested in a pilot randomised controlled trial (RCT), primarily to evaluate the feasibility and additionally the potential efficacy.

**Methods/design:**

*Design*: Pilot RCT with intervention and control (usual care) arms plus mixed-methods process evaluation. *Participants*: 100 ICU patients (50 per arm) of three ICUs in a university hospital (Regensburg, Germany), ≥ 18 years with an ICU stay of > 5 days, a sequential organ failure assessment (SOFA) score > 5 during the ICU stay and a life expectancy of more than 6 months.

*Intervention*: The intervention will contain three components: information, consultation and networking. Information will be available in form of an intensive care guide for patients and next of kin at the ICU and phone support during follow-up. For consultation, patients will visit the ICU follow-up clinic at least once during the first 6 months after discharge from ICU. During these visits, patients will be screened for symptoms of PICS and, if required, referred to specialists for further treatment. The networking part (e.g. special referral letter from the ICU follow-up clinic) aims to provide a network of outpatient care providers for former ICU patients. *Feasibility Outcomes*: Qualitative and quantitative evaluation will be used to explore reasons for non-participation and the intervention´s acceptability to patients and caregivers. *Efficacy Outcomes*: Health-related quality of life (HRQOL) will be assessed as primary outcome by the physical component score (PCS) of the Short-Form 12 Questionnaire (SF-12). Secondary outcomes encompass further patient-reported outcomes. All outcomes are assessed at 6 months after discharge from ICU.

**Discussion:**

The PINA study will determine feasibility and potential efficacy of a complex intervention in a pilot RCT to enhance follow-up care of ICU survivors. The pilot study is an important step for further studies in the field of ICU aftercare and especially for the implementation of a pragmatic multi-centre RCT.

**Trial registration:**

ClinicalTrials.gov, NCT04186468. Submitted 2 December 2019

**Supplementary Information:**

The online version contains supplementary material available at 10.1186/s40814-021-00796-1.

## Background

Due to medical progress the number of intensive care unit (ICU) survivors increased over the past decades in industrial nations [1, 2]. As a result of the ICU stay, ICU survivors often suffer from cognitive, physical and mental impairments, also known as post-intensive care syndrome (PICS) [3–5]. Epidemiological data from international studies on PICS in general are very scarce. However, it is assumed that one-half or more of former ICU patients will suffer from some component of PICS after discharge from ICU [6–10]. Estimates of the incidence of muscle weakness in ICU survivors in the USA, for example, range from 20 to 80% [11]. 30–80% of patients have cognitive impairments and 10–50% suffer from post-traumatic stress disorders (PTSD) [12–14].

These impairments are associated with a higher utilization of medical services [15–17] and a reduced (health-related) quality of life (HRQOL) [18, 19]. In addition, former ICU patients often show up in aftercare as multi-morbid patients and it has been shown that the provision of continuity of care for multi-morbid people is a special challenge [20]. This is one of the reasons why specific follow-up services are recommended for these patients [21].

There are various aftercare models potentially addressing PICS [22, 23], including ICU follow-up-clinics [24]. Follow-up services in general vary, for example, in the way they are managed (e.g. led by nurses or intensivists) [25], the type of consultation (e.g. conducted face-to-face or by telephone) or the number of consultations (e.g. weekly or monthly). They may also differ in the criteria used to select patients (e.g. all ICU survivors or stratified risk groups). Even within the concept of ICU follow-up clinics there currently exists no uniform concept, which makes it difficult to assess effectiveness [26]. Thus, there is only some evidence of little or no positive effects on mortality, HRQOL, PTSD or depression. No negative effects of ICU follow-up clinics are reported. In the UK, the first ICU follow-up clinic was established in 1985 and by 2006 almost every third ICU was linked to an ICU follow-up clinic [27]. Also in the USA and Scandinavian countries, various models of ICU follow-up clinics or services exist [24, 26, 28], but none in Germany so far.

Following the recommendations for the development and evaluation of complex interventions [29], we formed a multi-disciplinary stakeholder group composed of researchers and health care professionals to develop a concept for a complex intervention in ICU aftercare, based on existing literature and extensive qualitative research with health care professionals, ICU survivors and their next-of-kin. The primary objective of this pilot study is to evaluate whether this concept of a pragmatic randomised controlled trial (RCT) on the effects of an ICU follow-up clinic is feasible in terms of recruitment, randomisation, intervention and follow-up. Additionally, we want to explore if the ICU follow-up clinic itself shows effects in improving physical HRQOL of ICU survivors after a prolonged ICU stay (> 5 days) and use the results for sample size planning for the future pragmatic trial.

## Methods/design

Reporting of this study is based on the SPIRIT Checklist, a guidance for content of clinical trial protocols [30, 31].

### Scientific hypothesis

A pilot study of a pragmatic RCT on the effects of an ICU follow-up clinic shows to be feasible in terms of recruitment, randomisation, intervention and follow-up. The pilot study indicates improved physical HRQOL of ICU survivors being treated at the ICU follow-up clinic.

### Trial design

**Fig. 1:**
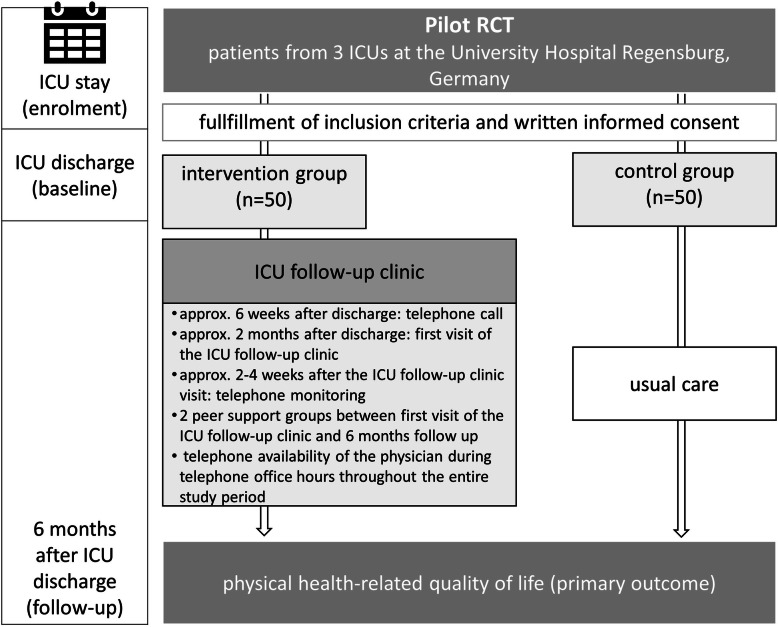
Overview of the study design, participant flow and a coarse description of the intervention components

**Fig. 2:**
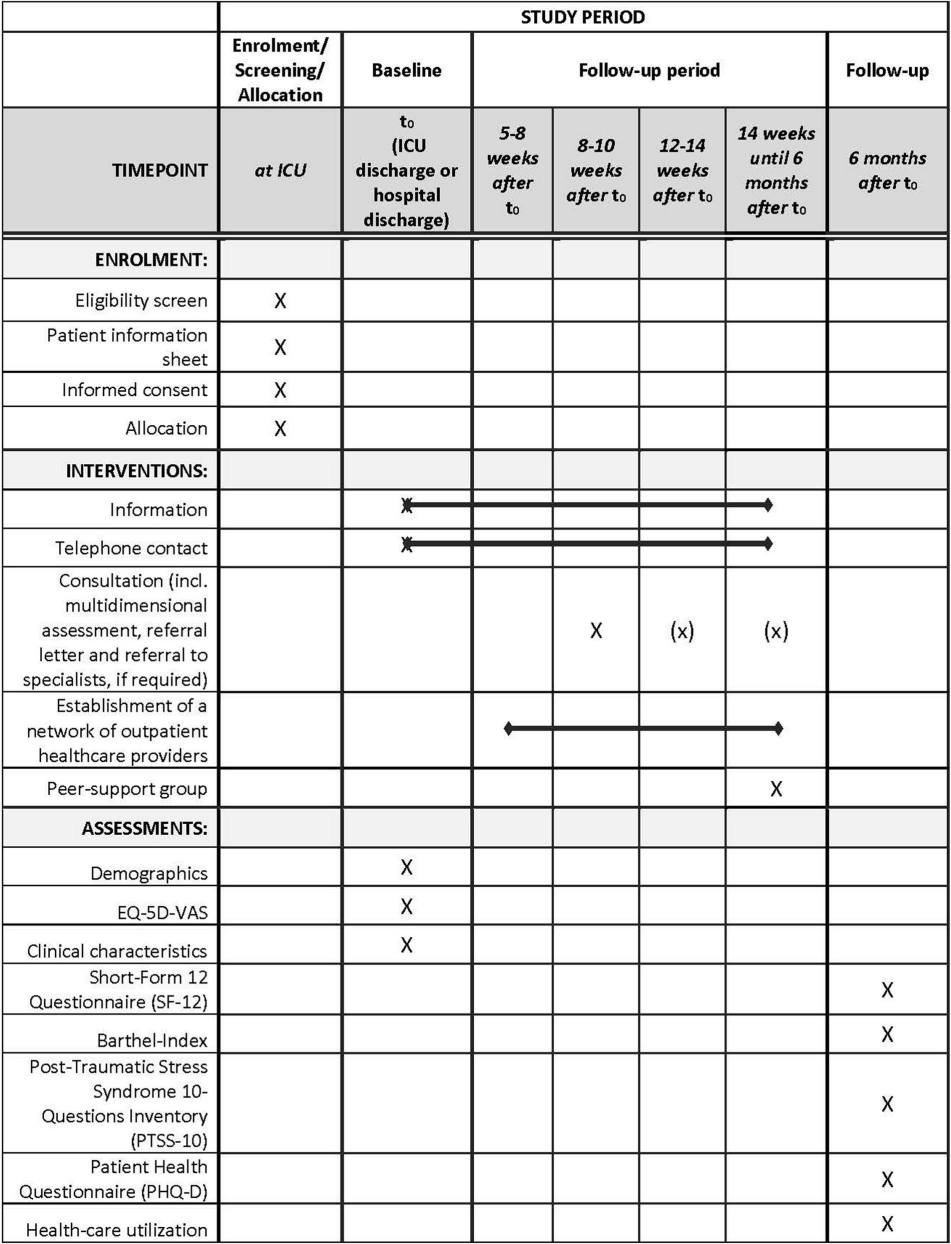
Schedule of enrolment, interventions, and assessments based on the SPIRIT recommendation

This study is a pragmatic, single-centre, superiority, two-armed pilot RCT to determine feasibility of a future trial comparing usual care with an ICU follow-up clinic. To explore acceptability and feasibility, we will also conduct a mixed-methods process evaluation as part of the pilot RCT. Additionally, as basis for sample size planning for the future trial, we want to assess patient reported outcomes to investigate potential efficacy. Figure 1 shows an overview of the study design and data collection procedures. Figure 2 reports the schedule for enrolment, interventions and assessment according to the SPIRIT template. The pilot trial started in December 2019 and is scheduled to last until October 2020. Patients will be followed-up for 6 months after ICU discharge.

### Participants

**Fig. 3:**
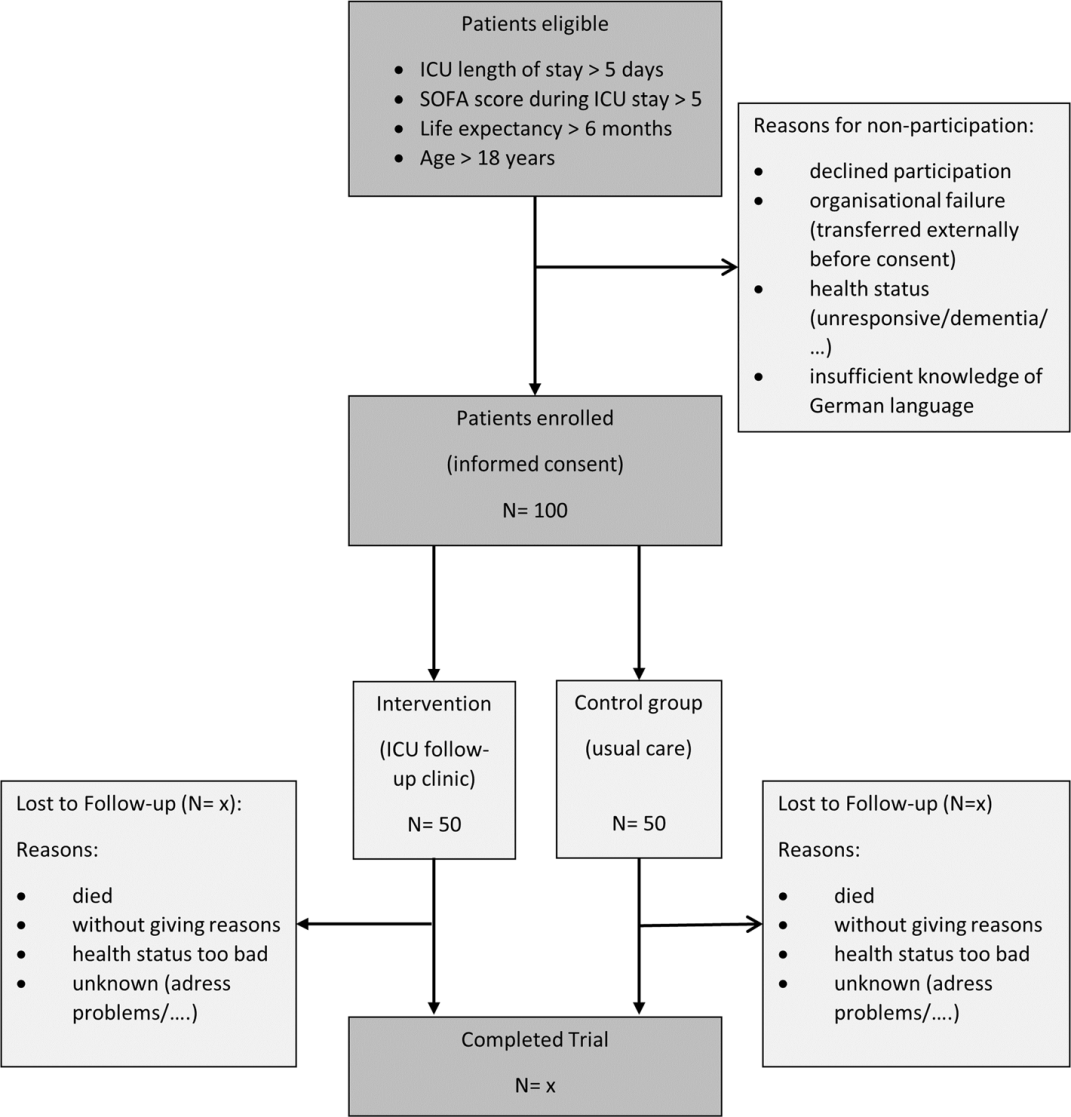
Planned participant flow in the PINA study

We will recruit participants meeting the inclusion criteria from three intensive care units (two medical and one surgical ICU) at the university hospital Regensburg, Germany. There will be 50 participants each in the intervention and control group. Eligible patients are screened on a daily basis by the ICU follow-up clinic team (study physician and nurse). Patients will be approached at the end of their ICU stay and will receive no incentive. The physician will explain the pilot trial, hand out the study information flyer and will be available to answer questions. Written informed consent will be obtained at the end of the ICU stay or shortly afterwards, when patients are transferred to a normal ward. Figure 3 illustrates the participant flow in the presented study. If the selected participant refuses to participate in the study, we will record reasons for non-participation.

### Inclusion criteria

Patients will be eligible to participate if they meet the following criteria: 18 years or older, duration of ICU stay more than 5 days, SOFA (sequential organ failure assessment) score greater than five at any time of the ICU stay and expected survival time greater than 6 months estimated by intensivists.

### Exclusion criteria

Patients will be excluded if they are less than 18 years old, gave no written informed consent (unable or unwilling), are not expected to survive 6 months after hospital discharge, are unable to complete questionnaires or have insufficient German language skills.

### Sample size

We chose a total of 100 participants for the pilot study, i.e. 50 per arm. This sample size is expected in literature to allow for a detailed analysis of feasibility and to be sufficient for an estimation of the difference in HRQOL (continuous variable) between intervention and control group which will be used for sample size estimation for the future pragmatic trial [32, 33].

### Randomisation

We will use computer-generated permuted block randomisation with blocks of size 10 to ensure balance between groups over short time spans, such as shifts and days of the week, as well as over the entire course of the study [34]. Treatment assignments (ICU follow-up clinic, usual care) will be placed in opaque, sequentially numbered envelopes prepared by the study team who has no contact with participants. The responsible study physician will only know the assignment after informed consent of the participant. The study physician will inform the participant about the result of the randomisation. Blinding of the study physician or participants after randomisation will not be possible. However, the research team performing data analysis will be blinded with regard to the participant’s allocation to intervention or control group.

### Intervention

The intervention was developed in a participatory process. The participatory development process of the intervention included one-on-one interviews with former intensive care patients (*n* = 26) and with next of kin of former intensive care patients (*n* = 23, not necessarily belonging to the interviewed patients). Six focus group discussions (*n* = 41, average duration of 97 min) and six expert interviews were conducted to capture also the perspective of the health care professionals. In total, persons from nine different professions were interviewed, with one third of the participants being physicians, followed by nurses (23%) and physiotherapists (17%). In addition, a short online survey with 15 items was sent to the health care professionals (n=46).

This information was evaluated, summarised and integrated with evidence from the literature and evidence from claims data analysis to create a first draft of the intervention [35]. The further development of the intervention was then carried out in 2 workshops with 23 and 21 participants each, composed of different health care professionals (physicians, nurses, therapists) and scientists (involved authors of the paper: MR, SB, CB, CF, VB, MM, CA).

The first workshop was used for a rough conceptualisation based on intervention mapping, while the second workshop was used for further specification of the concept according to the design-thinking approach. The results of both workshops were distributed to all participants and interested parties for correction. The concept was then finalised and written down by the interdisciplinary project team consisting of physicians, scientists and nurses. This extensive development process was assumed to possibly increase the effectiveness of the intervention.

**Fig. 4:**
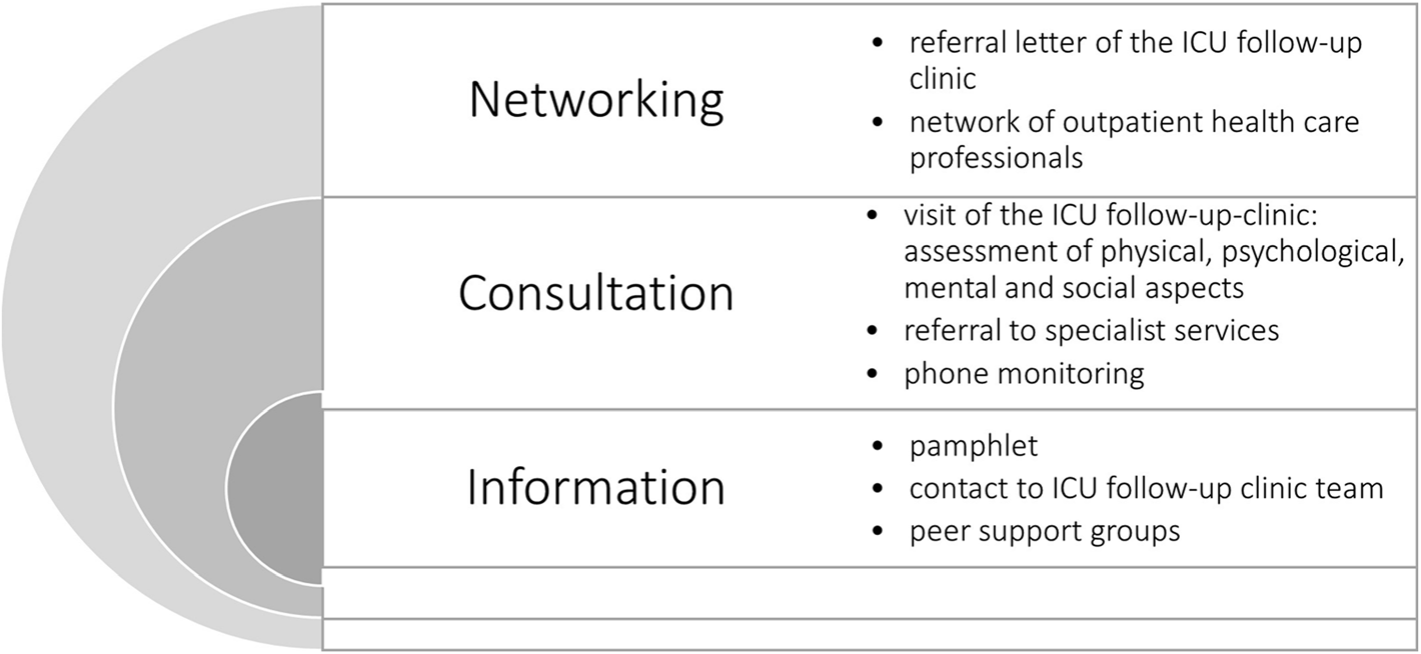
Main components of the ICU follow-up clinic

The intervention will contain three main components: information, consultation and networking (see Fig. 4). The design and timing of the appointment(s) has been determined by focus group interviews and an online survey with health care professionals. Information to participants will be provided by a pamphlet (developed by ICU steps [36] and translated by Deutsche Sepsishilfe and *German* Society of Skilled Nursing and Functional Services e.V. (DGF) [37]). Further, patients will have the possibility to contact the ICU follow-up clinic team by telephone at any time during the intervention. Study participants and their next of kin will be encouraged to join two self-help group meetings. A self-help group meeting will include a short information talk on a topic that has previously been shown in the consultations in the ICU follow-up clinic to be important to most participants. The lecture will be given by a member of the ICU follow-up clinic or by an outpatient health care professional, depending on the selected topic. Afterwards, both the participants and their next of kin will have the opportunity to get to know each other and to share their experiences. Patients and their next of kin will meet in separate rooms. This is assumed to facilitate disclosure of possibly difficult situations patients or next of kin are experiencing during the caregiving process.

Consultation will be provided in appointments at the ICU follow-up clinic at least once at about 2 months post-ICU discharge: initially, a checklist will be completed to assess physical, mental, cognitive and social functioning. The checklist will be based on standardised questionnaires (Mini-Cog [38], PHQ-8 [39], GAD-7 [40], PTSS-10 [59]), physical examination (chair rise test [41] and hand grip strength [42]), medical check-up (including blood pressure and body weight) and structured questions asked by the study physician (including symptoms of dysphagia and neuropathy). Results from this screening checklist will be used by the study physician of the ICU follow-up clinic to guide further treatment and thus to select the appropriate medical specialties, for example. Later, participants and next of kin will have the opportunity to discuss their ICU experience and to visit the ICU. This procedure has the potential to prevent posttraumatic stress by reintegrating traumatic memories. Where indicated, participants will be referred to outpatient medical specialists and/or outpatient therapists (ergotherapists, psychotherapist, logopedics, physiotherapists). After each clinic visit, a referral letter of the ICU follow-up clinic will be written primarily to the treating general practitioner and discussed with the participant. All intervention treatments will use the standard health care system processes. If the participant is not mobile, the ICU follow-up clinic team will visit him or her at home. All parts of the assessment can also be conducted at the patients’ home. Only the study physician’s letter and any referrals are then sent to the patient by mail in case of a home visit. Following the appointment, participants will be monitored by phone (one or more times depending on their needs) by the study physician. The physician will first explore the patient’s general state of health and then will ask to what extent the recommended interventions have been accepted and implemented. Any physical, mental or cognitive impairment will be inquired according to several items addressed during the visit in the ICU follow-up clinic. Based on monitoring results, participants may be encouraged to visit the ICU follow-up clinic once more or to contact their general practitioner. The third component concerns the establishment of a network of health care professionals who agree to care for participants of the ICU follow-up clinic or to provide information to other health care professionals. Since PICS can affect all health-related domains of former intensive care patients, the members of the network should represent different professions, disciplines and health care sectors. The referral letter of the ICU follow-up clinic is also intended to promote cooperation with the physicians and therapists providing further treatment.

### ICU follow-up clinic personnel

The ICU follow-up clinic will be led by a physician (ICU specialist) with support from an intensive care nurse. The nurse will coordinate the appointments, e.g. via telephone calls, and will take care of collecting the questionnaires. The physician will provide medical information during the ICU stay in present and via telephone during the follow-up period. They will focus on consultation, which will include interpreting the questionnaires, assessing physical, mental and social functioning and the referral to specialists. To improve adherence to the intervention, both a study manual and an intervention manual will be provided to all who are involved. The manuals contain the following information (Table 1):

**Table 1:** Overview of the contents of the study and intervention manual

Study manual	Intervention manual
• Recruitment • Inclusion and exclusion criteria • Patient information and consent • Baseline and follow-up survey • Randomisation • Data extraction and transmission • Description of the outcome measurements • Data analysis • Process evaluation • Scientific output	• Guidelines for participant contacts (informed consent conversation, telephone monitoring) • Description of intervention materials (e.g. flyer, pamphlet) • Description of the organisational context of the intervention (e.g. premises and consultation hours of the outpatient clinic) • Template for the medical letter of the ICU follow-up clinic visit • Key features for planning the patient support group

### Control treatment

Participants in the control group will receive the usual care without any additional information or consultation (no intensive care follow-up after hospital discharge), since there are not any ICU follow-up clinics in Germany. In addition, there is insufficient evidence on the effectiveness of ICU follow-up clinics in other countries; Schofield-Robinson et al. therefore propose to consider only the ICU follow-up clinic compared to standard care [24].

### Measures/outcomes

#### Participant characteristics

At baseline, sociodemographic data (e.g. age, gender, socioeconomic and marital status) as well as disease- and therapy-related characteristics will be extracted from the patient data management system. Disease-related characteristics include reason for admission to the ICU, as well as main and secondary diagnoses. Furthermore, the severity of disease will be captured by the Sequential Organ Failure Assessment (SOFA) score and the Simplified Acute Physiology Score (SAPS). Therapy-related characteristics will include characteristics of the ICU stay (e.g. duration, invasive ventilation and use of extracorporeal life support). HRQOL at baseline will only be assessed in form of the EQ-5D Visual Analogue Scale (VAS) [43] in order to minimise the burden on the participants during the acute phase of disease.

### Feasibility outcomes

**Fig. 5:**
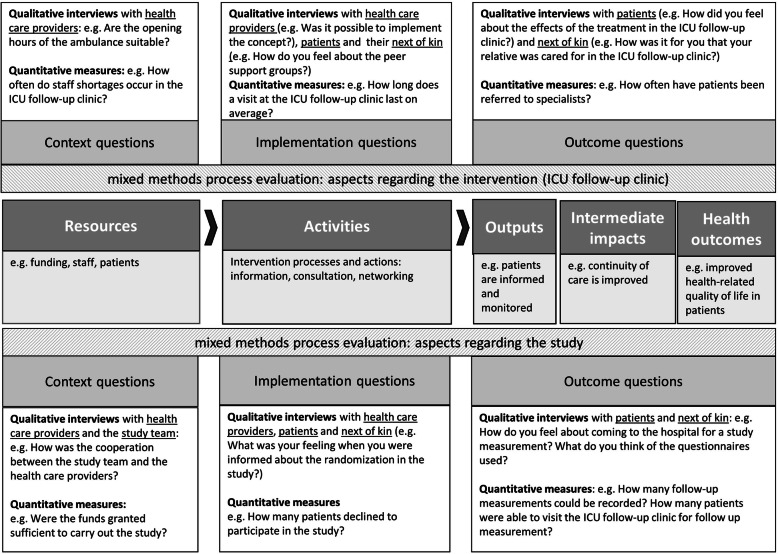
Overview of the components of the process evaluation in the PINA study according to the logic model

We will include process evaluation to explore aspects of feasibility and acceptability of both the trial procedures and the intervention. We will explore aspects of implementation, namely how intervention delivery is achieved and what is delivered (including dose, fidelity, reach and adaptions) [29]. Furthermore, we will investigate mechanisms of impact, as well as unanticipated pathways and consequences. We will use a logic model (Fig. 5) to frame our evaluation questions [44].

The process evaluation will include qualitative and quantitative components [45]. Figure 5 shows an overview of the process evaluation components and contains exemplary questions. The implementation of the trial and the intervention are influenced by context factors, which will also be documented and considered in the evaluation. The quantitative data will be collected during the intervention period, focusing on intervention fidelity and dose, and will be evaluated by frequencies. This will include, for example, the calculation of recruitment and dropout rate. Furthermore, after each visit, the study physician or nurse will take notes on characteristics of the visit in a standardised observation sheet. Acceptability of the study as well as of the intervention is in part also quantitatively recorded (e.g. via response rates). The qualitative data will be collected at the end of the intervention. Semi-structured one-to-one interviews will be conducted with health care professionals who delivered the intervention, participants (control and intervention group), next of kin and outpatient health care professionals, who will be involved in the treatment of the participants. In some circumstances, paired interviews (participants and next of kin) may be appropriate. We aim to conduct qualitative interviews with 10 participants in the control and the intervention group, respectively. In order to achieve heterogeneity among the participants in the interviews, we will purposively contact participants with different sociodemographic and disease related characteristics (e.g. age, sex, severity of disease). A researcher with qualitative research experience will conduct the interviews. The researchers are part of the PINA study team. We will design a topic guide to explore the following aspects: general opinions towards the study including acceptability and the willingness to undergo randomisation (all participants), general opinions towards the intervention, views on the intervention components, facilitators and barriers for attendance and adherence, the perceived impact of the intervention and possible improvements (intervention group). All interviews will be audio recorded, transcribed verbatim and de-identified with regard to the identity of the interviewee and mentioned clinicians. Content analysis will be performed using a computer-assisted qualitative data analysis software package (Atlas.ti).

In order to evaluate feasibility, we will consider, e.g. consent rate, attrition rate, adherence rate to the intervention, percentage of missing values in connection with the results of the qualitative interviews on feasibility and acceptability of the study and its implementation. We have deliberately decided against certain thresholds because there will not be a single threshold above which the RCT is not feasible anymore. Rather, our intention is to adapt individual parts of the pilot study to make a later large-scale study possible.

### Potential efficacy outcomes

#### Primary outcome

Primary efficacy outcome will be the physical health-related quality of life at 6 months after informed consent/ICU discharge. We chose this outcome as primary outcome because physical impairments are very common at 6 months. For example, a study from the UK reported mobility problems 6 months after the ICU stay in 64% of ICU survivors [46]. A prospective multi-centre study in Germany also showed that the limitation of the physical component is more pronounced at 6 months compared to the mental component [47]. Physical health-related quality of life will be assessed by the physical component scale (PCS) of the Short Form-12 self-report questionnaire (SF-12) [48]. This comprehensive, generic questionnaire comprises overall twelve items resulting in a physical (PCS) and mental component scale (MCS, see secondary outcomes), which will be scored according to published algorithms (German norm values; resulting in a standard score with mean = 50 and standard deviation = 10) [49]. Scores can range between 0 and 100, with higher values indicating higher HRQOL. The questionnaire has been used in critically ill patients before [23, 50], takes only minutes to complete and can be self-completed or interviewer assessed. Psychometric properties have not been tested in former critically ill patients so far, but studies on validity and reliability of the SF-12 in populations of older patients, which also applies to most former intensive care patients, show acceptable measurement characteristics [51, 52]. In our study we will use the German translation with a 1-week recall period [53].

#### Secondary outcomes

Secondary efficacy outcomes focus on the most relevant sequelae of ICU patients and encompass physical, mental and social impairments. The mental component summary score (MCS) of the SF-12 questionnaire will be the first of our measured secondary outcomes. Activities of Daily Living (ADL) will be assessed by the Barthel-Index [54, 55], which evaluates ten everyday functions. The degree of independence or care dependence can be assessed on a score ranging between 0 (complete need for care) and 100 points (independence). The Chair Rise Test [41, 56] and measurement of the hand grip strength will be used to assess participants‘ physical functioning and muscular strengths. In the Chair Rise Test, anyone who cannot get up from a chair at normal height five times in 11 s or less without supporting himself with his arms is considered to be at a higher risk of falling. The hand grip strength as an indicator of overall muscle strength will be assessed using a digital dynamometer (Jamar Plus+ Digital Hand Dynamometer) [42, 57]. We will measure the grip strength of both hands and use the maximum of these values for comparison with the standard values of Dodds et al. [58] stratified by age and sex expressed in percentiles, mean and standard deviation. The assessment of psychopathological symptoms comprises anxiety and panic disorder, depression and post-traumatic stress disorder (PTSD). PTSD is measured by the German translation: (Maercker, A. (1998) Posttraumatische Stress Skala-10 (PTSS-10) – deutsche Version modifiziert nach Schüffel u. Schade, unpublished manuscript, Universität Zürich, Klinische Psychologie II) of the Post-Traumatic Stress Syndrome 10-Questions Inventory (PTSS-10) [59], which consists of two parts. The first part assesses memories of traumatic experiences during the ICU stay (e.g. nightmares) and the second part measures the intensity of ten PTSD symptoms (e.g. emotional numbing) experienced presently by the participants. Each symptom is rated from one (never) to seven (always). A total score of more than 35 predicts a likely diagnosis of PTSD [60]. The other psychopathological symptoms will be measured by using modules from the German version of the Short Form of the Patient Health Questionnaire (PHQ-D) [61]. This brief screening instrument with 15 items is designed to establish DSM-IV (Diagnostic and Statistical Manual of Mental Disorders) [62] criteria-based psychiatric diagnoses. We will use the questions on depression and anxiety and panic disorders. The extent of ambulatory and stationary health care use among the former ICU patients will be assessed by self-reported contacts with health services using a questionnaire. In addition, HRQOL of next of kin will be assessed using the SF-12 questionnaire (MCS and PCS).

### Outcome assessment

Outcomes will be assessed at 6 months after discharge from ICU. Study participants will be asked to visit the study centre for outcome assessment. Trained study personnel will hand out self-report questionnaires, provide standardised instructions and perform physical measurements. Missing data will be minimised by having study personnel available at all times to check the completeness of the questionnaires or to answer participants´ questions. If participants cannot visit the study centre, home visits will be scheduled. If a participant discontinues the trial before outcome assessment, only the baseline data and the reasons for discontinuation are included in the analysis.

### Data analysis

**Fig. 6:**
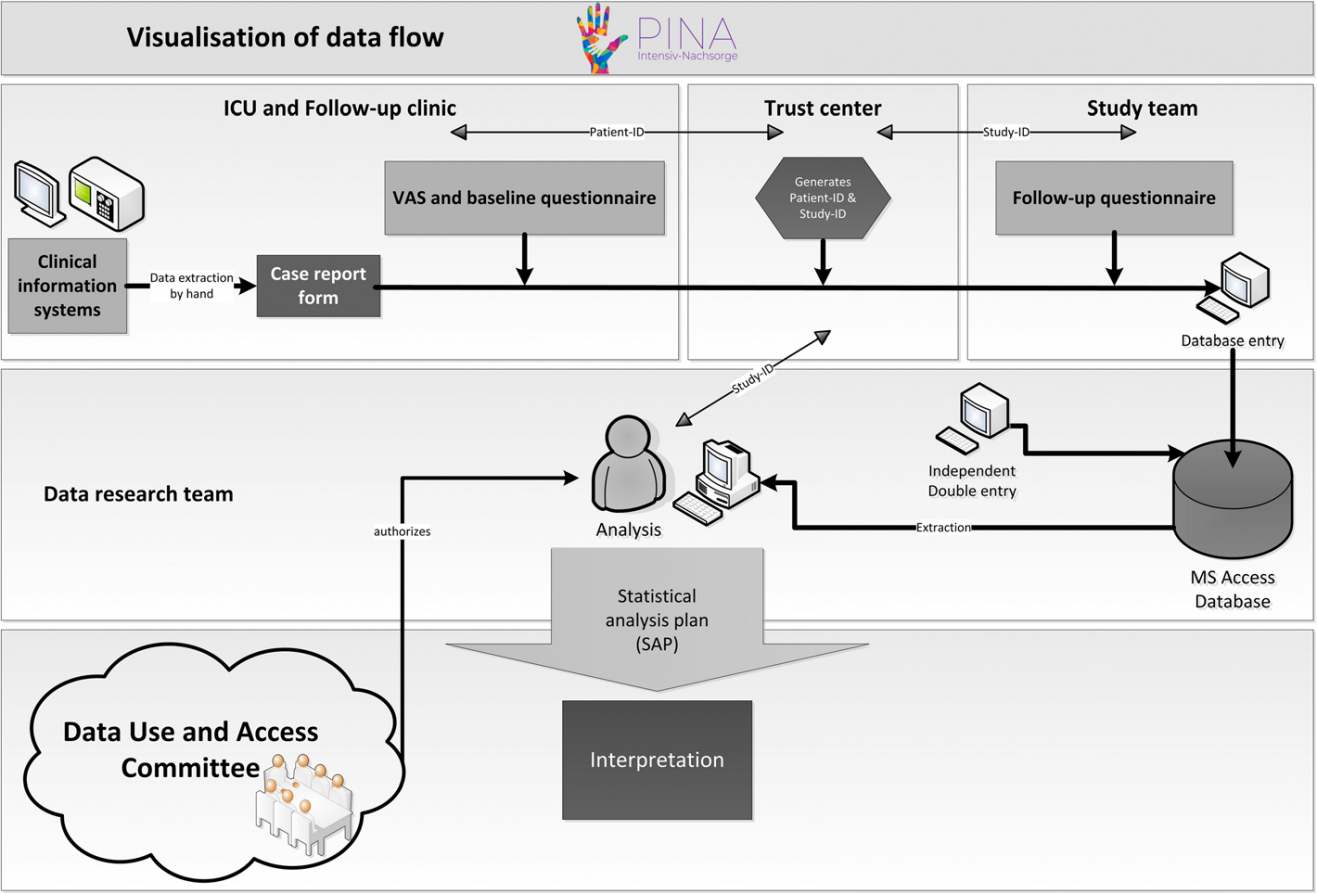
Flow of study data in the PINA project

A Data use and Access Committee, consisting of the three consortium partners, is part of our study, which will monitor the entire planned use of the data and releases it for analysis (see Fig. 6). We will write a statistical analysis plan before doing any data analysis. Group allocation will be masked by our trust centre during analysis of the primary outcome. The treatment effect (ICU follow-up clinic) on HRQOL as primary outcome and the secondary outcomes will be assessed using analysis of covariance according to the intention-to-treat-principle. Thus, all randomly assigned participants will be included in a complete case analysis. Multiple imputation will be considered for missing follow-up data as part of sensitivity analysis [63]. Descriptive statistics will be used to determine participant characteristics and to check their distribution at baseline in the intervention and control group. For sensitivity analysis, a per-protocol analysis will be performed additionally. The primary outcome physical HRQOL will be compared 6 months post randomisation. We will calculate point and interval estimates with the respective confidence intervals for the difference in medians. Secondary analysis will be performed depending on scale level and for descriptive purposes only. No formal hypothesis testing will be performed.

### Ethical principles and description of risks

The institutional Ethics Committee of the University of Regensburg (19-1522-101) approved the study protocol. The study is planned and conducted in accordance with the declaration of Helsinki, the medical professional codex, the European General Data Protection Regulation (DS-GVO) and the Federal Data Protection Act (BDSG). Participants (and next of kin) will participate voluntarily and will give written informed consent. Participants will be informed that they can cancel their participation at any time without disclosing reasons for their cancellation and without negative consequences for their future medical care. This also applies to participants in the intervention group who already made use of ICU follow-up services.

The risks for participants arising from participation in the study are generally considered low. No negative effects of ICU follow-up clinics are reported [24]. Therefore, there are no specific risks related to the intervention and thus no rules for stopping the intervention.

### Data collection, management and privacy issues

For study purposes, quantitative and qualitative primary data as well as clinical data will be collected. In order to keep the burden on the participants as low as possible, as much data as possible will be extracted directly from the hospital records (contact information, disease- and therapy-related characteristics, sociodemographic characteristics). More detailed information on sociodemographic characteristics will be obtained directly from the participants. Participants themselves will provide information on the primary outcome physical HRQOL at baseline and follow-up. At follow-up, further patient-reported outcomes are recorded as well as self-reported health care utilisation and physical functioning (e.g. Chair Rise Test). HRQOL of next of kin will also be recorded at follow-up. Personal information will only be collected on the consent form. These will be stored in the Trust Centre in locked cabinets without access by unauthorised persons.

All data will be handled according to the Medical Confidentiality Rules and German Federal Data Protection Act (BDSG), as well as the European General Data Protection Regulation (GDPR/DS-GVO) and all subordinate acts and ordinances. Except for contact information required for phone monitoring and home visits, all data will be recorded and stored pseudonymously. Person-identifying data will be handled by a trust centre and stored separately from study data and can only be linked via a separately stored pivot table on a PC without access to the internet. The chances that an illicit link can be made between the data are very small in light of the precautions we have taken. Therefore, the risk of an unauthorised de-pseudonymisation of the study participant’s data is considered extremely low (see recital 75 DS-GVO). In this context, we have drawn up a comprehensive data protection concept. All study related data and documents will be stored on protected servers of either the University of Regensburg or the University of Magdeburg. Only members of the study team will have access to the respective study files. Data exchange between the study team and the outcomes assessor will be via a secure platform where study data can only be accessed via double authentication (username/password for login and additional password to access the database). We have summarised the data flow schematically in Fig. 6.

### Amendment to protocol

Due to the COVID-19 pandemic, the Bavarian authorities ordered restrictions on research activities at all study centres. Therefore, all activities related to recruitment, inclusion of participants, the work of the follow-up clinic as well as the follow-up survey had to be discontinued on 20 March 2020. As a result, we needed to plan for a restart of the RCT. Since the duration of the interruption was not foreseeable at this time, there was initially no other choice than to completely restart this pilot RCT afterwards. In a RCT, differences between the intervention group and the control group should be determined. However, if a part of all patients were treated before the pandemic and another part of the patients were treated during or even after the pandemic, these patients cannot be compared with each other anymore, as the care provided and the circumstances repeatedly changed. Furthermore, it was not foreseeable whether and how the intervention (ICU follow-up clinic) could have been carried out. After intensive consultations among the study team, with the hospital management and the funding body, it was decided to reduce the number of cases to 40 participants. This measure had to be taken mainly for research economic reasons. The limited duration of the project and limited financial resources did not allow a further extension of the project to recruit another 100 patients. We were able to resume activities on 15 June 2020 and believe that even with this reduced number of study participants, our overall aim to evaluate feasibility and effects of an ICU follow-up clinic can still be achieved. Study completion is expected in May 2021.

## Discussion

To our knowledge, PINA is the first study evaluating the feasibility and preliminary effects of a complex intervention in form of an ICU follow-up clinic for ICU survivors in Germany. Based on the key elements of development and evaluation of complex interventions using the framework of the medical research council (MRC) [29], this study concerns mainly the phases *development*, *feasibility and piloting*.

We have invested significant resources in the *development* of the intervention which was done by a participatory process in which patients, next of kin and health care professionals were involved. To be more precise, focus groups with health care professionals and one-to-one interviews with experts from the PICS field were conducted. At the same time, face-to-face interviews with patients and with next of kin were conducted. The results of these qualitative research projects were then further elaborated into a final concept in several workshops.

The implementation of the complex intervention in the *feasibility and piloting phase* aims at testing procedures, estimating retention and determining the sample size. In spite of the sample size being too small for a full effectiveness evaluation, we will determine effectiveness preliminarily, thus anticipating the full-scale *evaluation phase* in a large pragmatic trial in the future. The implementation will be monitored by process evaluation to provide insights into unexpected or unanticipated consequences or to show why the intervention works. Depending on the evaluation and feasibility outcomes, the next steps will be either to modify the intervention and repeat the piloting phase or to proceed to implement the intervention on a large scale and test it in a pragmatic real-world RCT.

### Limitations

Even though the randomisation process may have been conducted correctly using appropriate randomisation strategies, a balanced distribution of known and unknown confounding factors (such as sociodemographic or clinical factors) between intervention and control group might not be achieved. Therefore, we will compare baseline characteristics of participants in the intervention and the control group. The follow-up period may be too short, and the effects of the intervention might appear only after the follow-up period of 6 months. A minimal spillover effect, e.g. due to the information about the study, cannot be completely ruled out.

### Strengths

The study introduces the first ICU follow-up clinic in Germany and thus tries to improve the aftercare of ICU survivors. The greatest advantage of the ICU follow-up clinic is its participatory development, which increases the likelihood of the intervention being effective. In addition, all intervention treatments will use the standard health care system processes, allowing the ICU follow-up clinic to be well integrated into the existing health care system in the future.

### Trial status and dissemination

The first participant was enrolled on 2 December 2019. Participants’ recruitment started at the time of submission of the study protocol and was completed during the review process. Results will be presented at national and international conferences and reported in peer-reviewed journals.

## Supplementary Information

**Additional file 1:.** SPIRIT 2013 Checklist: Recommended items to address in a clinical trial protocol and related documents

## Data Availability

Data can be requested from 1 year after study completion from the principal investigator.
